# Measuring the Association between Artemisinin-Based Case Management and Malaria Incidence in Southern Vietnam, 1991–2010

**DOI:** 10.4269/ajtmh.14-0461

**Published:** 2015-04-01

**Authors:** Corey M. Peak, Phung Duc Thuan, Amadea Britton, Tran Dang Nguyen, Marcel Wolbers, Ngo Viet Thanh, Caroline O. Buckee, Maciej F. Boni

**Affiliations:** Center for Communicable Disease Dynamics, Department of Epidemiology, Harvard School of Public Health, Boston, Massachusetts; Institute for Malariology, Parasitology, and Entomology, Ho Chi Minh City, Vietnam; Oxford University Clinical Research Unit, Wellcome Trust Major Overseas Programme, Ho Chi Minh City, Vietnam; Department of Global Health and Population, Harvard School of Public Health, Boston, Massachusetts; Centre for Tropical Medicine, Nuffield Department of Clinical Medicine, University of Oxford, United Kingdom

## Abstract

In addition to being effective, fast-acting, and well tolerated, artemisinin-based combination therapies (ACTs) are able to kill certain transmission stages of the malaria parasite. However, the population-level impacts of ACTs on reducing malaria transmission have been difficult to assess. In this study on the history of malaria control in Vietnam, we assemble annual reporting on malaria case counts, coverage with insecticide-treated nets (ITN) and indoor residual spraying (IRS), and drug purchases by provincial malaria control programs from 1991 to 2010 in Vietnam's 20 southern provinces. We observe a significant negative association between artemisinin use and malaria incidence, with a 10% absolute increase in the purchase proportion of artemisinin-containing regimens being associated with a 29.1% (95% confidence interval: 14.8–41.0%) reduction in slide-confirmed malaria incidence, after accounting for changes in urbanization, ITN/IRS coverage, and two indicators of health system capacity. One budget-related indicator of health system capacity was found to have a smaller association with malaria incidence, and no other significant factors were found. Our findings suggest that including an artemisinin component in malaria drug regimens was strongly associated with reduced malaria incidence in southern Vietnam, whereas changes in urbanization and coverage with ITN or IRS were not.

## Introduction

The most recent World Malaria Report from the World Health Organization (WHO) shows that between the years 2002 and 2012 malaria cases and deaths decreased by 10.4% and 28.4%, respectively, and widespread adoption of artemisinin-based combination therapies (ACTs) as first-line therapy for malaria is likely a significant contributor to these gains.^1^–^3^ In comparison to other antimalarial drugs, artemisinin is associated with reduced treatment failure rates,^4^ accelerated parasite clearance,^5^ and reduced mortality.^6^ Consequently, ACTs are the recommended treatment of both uncomplicated and severe malaria.^7^ In addition to improving patient outcomes, artemisinin is also active against certain stages of gametocytes, the parasite sexual stage whose mature forms are transmissible to mosquitoes.^8^ ACTs are therefore considered an important part of a concerted effort in many countries to achieve elimination through routine case management or mass drug administration strategies.^9^

The relative contribution of ACTs to reducing malaria transmission is difficult to assess, particularly in the context of changing population coverage with insecticide-treated nets (ITN) and indoor residual spraying (IRS). Since approval by the WHO in the early 2000s, ACT scale-up has frequently occurred in parallel with other transmission-reducing interventions, making it difficult to isolate the impact of each intervention independently.^10^–^14^ Although there have been few efforts to record changes in malaria incidence following ACT scale-up, select studies suggest that case management with ACT could play a critical role in reducing transmission of the parasite. A 1996 study of rapid replacement of mefloquine with ACTs in a refugee population in western Thailand reported a 47% reduction in *Plasmodium falciparum* infections over the 12-month follow-up.^15^ This study design was expanded to include local populations in the same region and documented a 34% reduction in *P. falciparum* cases over 6 years following investment in early case detection and treatment with artemisinin-based therapies.^16^ In Zanzibar, ACT introduction in 2003 was followed by no change in malaria admissions in 2004 and then a 68% reduction in 2005.^6^ Although the reported associations between case management with ACTs and malaria incidence vary in magnitude and reference treatment regimen, these studies suggest an appreciable population-level effect that needs to be further explored in pre-elimination settings.

In contrast to many malaria-endemic countries, Vietnam's centralized malaria control program enabled and documented a rapid shift from conventional drug regimens to those based on artemisinin. Vietnam was among the first countries to recommend first-line artemisinin use at a national level and annual records show a dramatic reduction in the burden of malaria during the years that artemisinin was introduced.^17^,^18^ Province-level data on artemisinin scale-up and changes in malaria case numbers in Vietnam present a unique opportunity to examine the association between case management with ACT and malaria transmission. Here, we examine data on malaria cases, prevention measures, antimalarial treatment, and improvements in health system capacity for 20 southern Vietnamese provinces over 20 years to estimate the impact of ACTs on malaria incidence between 1991 and 2010. In addition to annual data on all antimalarial drug purchase requests, provincial malaria control programs report data on the quantity of pyrethroid-group insecticides used for twice-yearly indoor residual spraying and bed net impregnation, approximate population coverage of ITN and IRS, and several general measures of the province's capacity to respond to high levels of malaria incidence. We show that artemisinin use is more strongly associated with a reduction in malaria incidence than other interventions including ITN and IRS.

## Materials and Methods

### Data.

**Figure 1.**
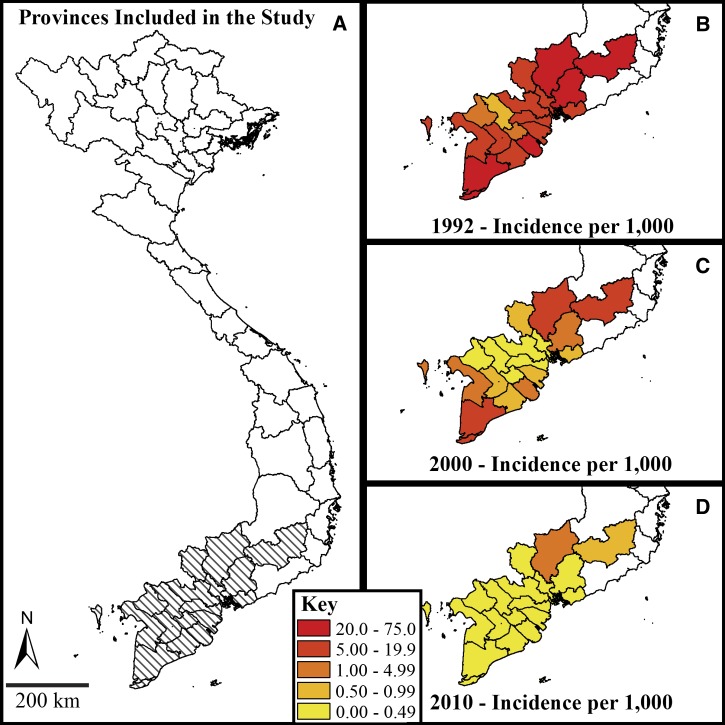
Vietnamese provinces included in the study are hatched (**Panel A**). Exponential decrease in annual incidence of suspected malaria cases per 1,000 persons is observed in the changes from 1992 (**Panel B**) to 2000 (**Panel C**) and 2010 (**Panel D**). Note the use of a non-linear scale to accommodate the large dynamic range in incidence.

The Institute for Malariology, Parasitology, and Entomology (IMPE) of southern Vietnam provided the data used in this analysis. Study provinces included An Giang, Bac Lieu, Ca Mau, Ben Tre, Binh Duong, Binh Phuoc, Ba Ria-Vung Tau, Can Tho, Hau Giang, Soc Trang, Dong Nai, Dong Thap, Kien Giang, Lam Dong, Long An, Tay Ninh, Tien Giang, Ho Chi Minh City, Tra Vinh, and Vinh Long. As a result of shifting administrative borders during the reporting period, certain study provinces were combined into groups: Binh Duong/Binh Phuoc; Bac Lieu/Ca Mau; and Can Tho/Hau Giang/Soc Trang. Malaria is a notifiable disease in Vietnam, and IMPE uses a standardized hierarchical reporting system to collect data on interventions and cases. These reports included counts of suspected malaria cases, slide-positive smears, smears positive for *P. falciparum* parasites, smears positive for *Plasmodium vivax* parasites, the quantities of each drug type ordered by provincial malaria controls programs, annual budgets, annual staff trainings, and the proportion of the population protected by ITNs or IRS. Records were translated into English and entered into an electronic database in collaboration with IMPE and the Oxford University Clinical Research Unit in Ho Chi Minh City, Vietnam. Annual reports from 1991 to 2010 were analyzed for the southern provinces according to the 2010 provincial boundaries (Figure 1A). The data set is structured as a yearly panel series for 20 provinces in southern Vietnam over 20 years.

The primary covariate of interest is the proportion of antimalarial drug regimens ordered by a given province each year that contain artemisinin. This proportion measure serves as a proxy for the probability that the treatment received by a malaria patient included artemisinin. We hypothesize that replacing non-artemisinin-based therapies with ACTs may reduce malaria transmission. Data on antimalarial treatment requests by provincial governments were available in the form of number of tablets for oral regimens and number of tubes for injectable regimens for the years 1992, 1994, 1995, 1997–2007, 2009, and 2010. Purchase requests are submitted to the national government in Hanoi by each province for anticipated treatment needs each year. The artemisinin-based antimalarial treatments documented in the reports include: injectable artesunate (60 mg tube); artemisinin (250 mg tablet); artesunate (50 mg tablet); dihydroartemisinin-piperaquine (40 mg dihydroartemisinin, 320 mg piperaquine tablet); and CV8 (32 mg dihydroartemisinin, 320 mg piperaquine, 90 mg trimethoprim, 5 mg primaquine tablet). The non-artemisinin based antimalarial treatments include: quinine-sulfate (250 mg tablet); quinine hydrochloride (500 mg tube); mefloquine (250 mg tablet); sulfadoxine-pyrimethamine (500 mg sulfadoxine, 50 mg pyrimethamine tablet); Fansidar (2.5 mL parenteral sulfadoxine-pyrimethamine tube); chloroquine (250 mg tablet); and primaquine (13.2 mg tablet). The reported number of pills and tubes were converted to standard regimens according to national treatment guidelines for a range of case types including child, adult, slide-confirmed *P. falciparum* infection, and slide-confirmed *P. vivax* infection. To impute missing data on antimalarial drug purchases for the years 1993, 1996, and 2008, estimates of the proportion of drug regimens containing artemisinin were imputed as a moving average of the year preceding and following the missing measurement. The main association was essentially unchanged in repeated analyses that exclude missing covariate information. Private sector drug purchases were not considered in this analysis because the vast majority of antimalarials in Vietnam are government manufactured for the national malaria control program and it is illegal to sell or prescribe these antimalarial drugs outside official provincial/national government clinics or hospitals. No data are available on private purchases, but it is likely that almost all malaria patients would go to a government clinic as treatment is free.

The proportion of individuals in a province protected by either ITNs or IRS was estimated as the sum of the proportions of individuals protected by ITNs and the proportion of individuals protected by IRS as reported by IMPE. This approach was chosen to conservatively overestimate distribution patterns known to include double-coverage of homes with ITNs and IRS. An individual was considered protected by ITNs if living in a household with at least one net per four household members.^13^ To impute missing data on IRS/ITN coverage, estimates were imputed as a moving average of the years preceding and following the missing measurement.

To account for changes to economic and developmental indicators in Vietnam from 1991 to 2010, we identified several variables potentially associated with changes in provincial health system capacity to control malaria. Candidate measures included counts of blood smears performed, counts of drugs purchased, budgetary figures, and staff training data. Measurement of the yearly count of blood smears performed was excluded as a potential explanatory variable because of the inability to distinguish between smears performed during active case detection and smears performed during general-population surveys. The total number of drugs purchased was strongly influenced by malaria incidence the previous year and could induce the observation of reverse causation. To avoid general and indirect metrics like gross domestic product, we chose to include two specific measures of health system capacity as covariates in the analysis by province and year: 1) the number of staff that received training in malaria case identification and management each year; and 2) the discretionary budget of the malaria control program, i.e., funds not allocated to purchasing or subsidizing antimalarials, bed nets, or insecticides. Yearly budget figures were adjusted for historical inflation in the Vietnamese Dong (VND)^19^ and converted to 2010 United States dollars (USD) for analysis; in 2010, 19,065 VND = 1 USD.

Population data stratified by urban and rural settlement type were provided by IMPE and the Vietnam Government Statistical Office. The proportion of each province's population considered to be living in an urban setting was calculated yearly and included in the analysis to account for the effects of urbanization on breeding and survival of *Anopheline* mosquitoes, the malaria vector. Yearly measurements from 1995 to 2007 were fit with a linear regression to impute missing values for this variable for the years 1991–1994 and 2008–2010 (Supplemental Figure 1).

### Statistical analysis.

Temporal trends of the primary covariates were evaluated for each province using non-parametric Spearman rank correlation tests to maximize power to detect changes over the study period and avoid misspecification. A simple linear regression of each covariate against time was fit to estimate the average annual change assuming a linear association. Significance thresholds for all tests were set to a 0.05 probability of type I error.

Annual malaria incidence was modeled with a Poisson regression model with the standard log link function. Generalized estimating equations (GEE) were used to measure population-averaged associations between covariates and malaria incidence after accounting for within-province correlation as a result of repeated observations over time.^20^ The working correlation structure between observations grouped in provinces was modeled as independent and standard errors were based on “sandwich” estimators robust to misclassification of within-group correlation structure and overdispersion.^20^,^21^ To fit the regressions, the *geepack* package in the statistical computing environment R was used.^22^–^24^

Predictors for four malaria incidence outcomes included 1) proportion of treatment regimens purchased that contained artemisinin, 2) the proportion of the population protected by either ITN or IRS, 3) the proportion of the population living in urban settings, 4) the number of staff trainings by provincial malaria control programs per 100 people, and 5) the discretionary malaria program budget per capita. Province population was used as an offset term to standardize annual malaria case counts and allow for the estimation of incidence rates. The impacts of intervention efforts on malaria transmission are assumed to occur within the year so associations between these predictors and the incidence response variables were measured in the same year.

## Results

### Malaria reduction in southern Vietnam.

**Figure 2.**
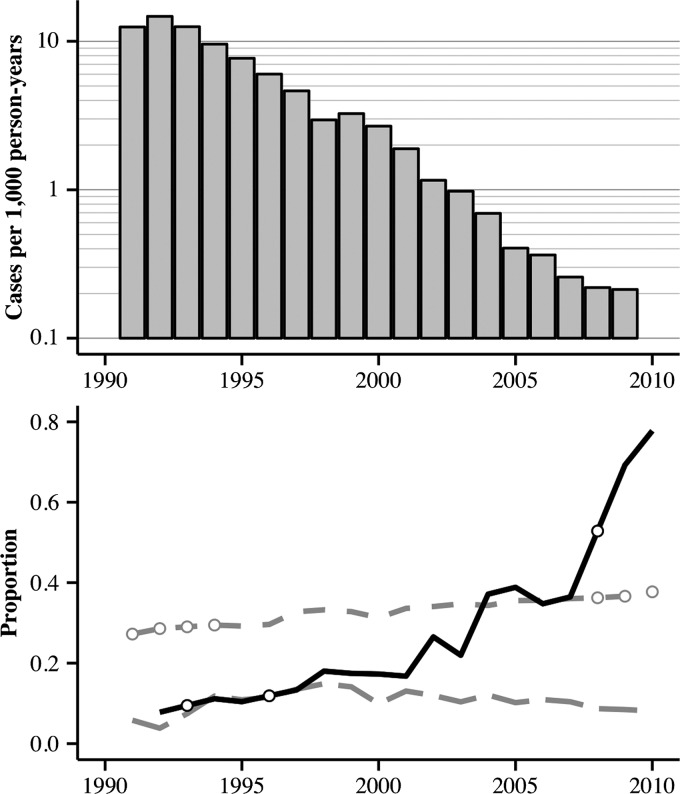
Annual incidence of suspected malaria across all study provinces is shown on the logarithmic scale in the top panel. The bottom panel shows annual proportions of drugs containing artemisinin (solid line), population living in urban settings (short-dashed line), and population protected by insecticide-treated nets or indoor residual spraying (long-dashed line). Open circles represent the value of imputed artemisinin and urbanization metrics for indicated years.

The total number of clinically diagnosed malaria cases in southern Vietnam decreased from 364,302 in 1992 to 73,146 cases in 2000 and 6,923 in 2010 (Figure 2). Severe cases declined even more steeply from 5,803 in 1992 to 239 in 2000 and 94 in 2010. A similar improvement in clinical outcome can be observed by the decrease in malaria deaths from 516 deaths in 1992 to 28 deaths in 2000 and 13 deaths in 2010. Due in part to increased access to ACTs, the case fatality ratio decreased by 85.9% (95% confidence interval [CI]: 75.6–91.8%) over the study period. The ratio of *P. falciparum* to *P. vivax* cases shifted from an average of 2:1 in the 1990s to 3:1 in the 2000s, although these changes were not uniform across the study provinces. See Supplemental Figure 2 for province-specific trends for the ratio of *P. falciparum* to *P. vivax* cases.

### Temporal changes in antimalarial interventions.

The percentage of purchased treatment regimens that contained artemisinin increased from 7.8% (province-specific range: 5.5–18.9%) in 1992 to 77.8% (province-specific range: 66.1–98.0%) in 2010 (Figure 2). Non-parametric Spearman rank correlation tests revealed this relationship to be strongly positive (ρ_s_ > 0.732) and highly significant (*P* < 0.02) in every province except Ba Ria-Vung Tau (ρ_s_ = 0.525; *P* = 0.057). Furthermore, a simple linear regression for each province captured an increase in the use of artemisinin between 3.3% to 6.5% in an average year (*P* ≤ 0.001) (Supplemental Table 1). This gradual preference for ACTs largely supplanted conventional regimens of chloroquine, sulfadoxine and pyrimethamine, and mefloquine in the region.

The percentage of the population reported to be protected by ITN ownership or IRS showed smaller and less consistent changes during this time period. Non-parametric tests report significant increases in coverage over time in Bac Lieu/Ca Mau, Tra Vinh, Ben Tre, and Can Tho/Hau Giang/Soc Trang; significant decreases in coverage over time in Ho Chi Minh City, Long An, Lam Dong, and Dong Thap; and no significant trends in the remaining eight provinces (Supplemental Table 2).

Non-parametric tests revealed a statistically significant but slow trend of urbanization over time in half of the study provinces, whereas no significant patterns over time were detected in the remaining half. Linear regressions sufficiently captured the trends identified in the non-parametric tests and, as described in the methods, these regressions were used to impute missing data for this covariate (Supplemental Table 3).

### Statistical modeling of malaria cases and antimalarial interventions.

**Figure 3.**
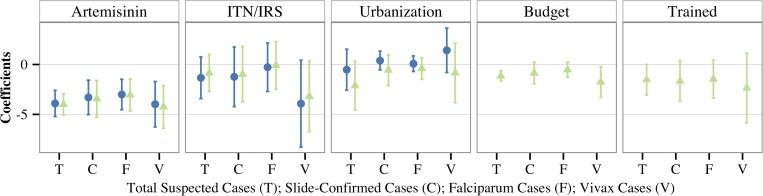
Regression coefficients and respective 95% confidence intervals are shown from models predicting each of the four malaria incidence outcomes: Total Suspected Cases (T); Slide-Confirmed (C); *Plasmodium falciparum* (F); and *Plasmodium vivax* (V). Blue circles represent results from models including the proportion of drugs containing an artemisinin component, proportion of provincial population covered by insecticide-treated nets (ITN) or indoor residual spraying (IRS), and proportion of provincial population living in urban settings. Light green triangles represent results from models with the addition of the following health systems capacity covariates: discretionary provincial malaria budget per capita and number of staff trained per 100 people.

Table 1 reports regression results from models including: 1) the proportion of drugs containing an artemisinin component, 2) the proportion of provincial population covered by ITN or IRS, and 3) the proportion of provincial population living in urban settings. The proportion of drug purchases containing artemisinin was negatively associated with each of the four malaria outcomes measured: 1) the number of clinically diagnosed suspected cases, 2) the number of slide-confirmed cases, 3) the number of slide-confirmed *P. falciparum* cases, and 4) the number of slide-confirmed *P. vivax* cases. Urbanization and coverage with ITNs or IRS were not significant predictors of any of the malaria outcomes after accounting for changes in the proportion of drug regimens that contain artemisinin (Figure 3).

Table 2 reports regression results from models expanded to include health system capacity metrics. A 10% absolute increase in the proportion of treatment regimens that contain artemisinin was associated with a 29.1% (95% CI: 14.8–41.0%) lower incidence of slide-confirmed malaria after accounting for changes in urbanization, ITN and IRS coverage, and selected indicators of health system capacity. Of the interventions tested, this association was the most robust to changes in model outcome including clinical malaria cases, slide-confirmed *P. falciparum* cases, and slide-confirmed *P. vivax* cases. The association between artemisinin/ACT use and malaria incidence was significantly protective for each province (Supplemental Figure 3), though the population-averaged results may be more appropriate for these data as they are highly correlated in space and time (see Supplemental Information).

Increases in the discretionary malaria budget, an indicator of health systems strengthening, were found to be associated with reduced malaria incidence. A 10% absolute increase in the discretionary provincial malaria budget per capita was associated with a 10.8% (95% CI: 6.0–15.4%) reduction in incidence of clinical malaria after accounting for changes in the proportion of drugs containing artemisinin, urbanization, ITN and IRS coverage, and malaria staff training. The second indicator of health systems strengthening, annual staff trained per 100 people, was marginally associated with reduced incidence of suspected cases (*P* = 0.052).

## Discussion

This analysis examines the association between adoption of artemisinin-based therapies and malaria incidence using a unique data set spanning two decades of malaria control where artemisinin scale-up was programmatically independent of changes to other interventions including ITN and IRS. As a result of centralized intervention implementation and recordkeeping, these data present an opportunity to study the impact of interventions that are typically distributed by several regional programs in a less coordinated fashion. These data record a nearly 10-fold increase in proportion of drug purchases containing artemisinin and a remarkable 98% decrease in annual malaria incidence over this 20-year period in southern Vietnam. This expansive range allowed us to observe a significant, negative association between case management with artemisinin-based therapy and malaria incidence while controlling for other primary malaria interventions. Of the interventions tested, this association was found to be robust to different measures of malaria incidence. Although urbanization and increased ITN/IRS use are known to reduce the burden of malaria, they are not found to be drivers of the dramatic changes in incidence that were observed in southern Vietnam during the 1990s and early 2000s, one of the reasons being that changes in these two measures were small compared with changes in artemisinin use; in 2010, 63% of the population of southern Vietnam were still living in rural areas. Our results support the hypothesis that case management with artemisinin-based therapies should have a substantial impact on transmission in addition to improving patient outcomes.

As Vietnam invests in the goal of national malaria elimination, the choice of end-game tools and strategies becomes increasingly urgent.^9^ Our results measure the association between key interventions and incidence in a region leading the country toward elimination. Although China to the north has set a national malaria elimination goal by 2020, Cambodia and southern Laos, which border southern Vietnam are further from elimination.^9^ Border provinces of Vietnam must therefore maintain intervention coverage to avoid a resurgence of incidence as experienced in the 1980s following deterioration of regional health systems.^17^ Of importance, the maintenance of key interventions including ITN and IRS coverage and replacement of non-artemisinin regimens with ACTs was sufficient to prevent resurgence during this two-decade study period. After controlling for these key interventions, provincial discretionary malaria budget was also found to have a significantly protective association with incidence of clinically diagnosed malaria and suggests an independent protective role of health sector capacity building.

Future analyses may consider applying these methods to provinces throughout Vietnam to increase generalizability and power to detect possible effects of other drivers of malaria case reductions. In particular, the effect of improved case management on malaria incidence is expected to be largest in settings like our study where transmission intensity is low, the proportion of malaria infections that develop symptoms is large, and treatment access is high.^25^

The primary covariate of interest, the proportion of purchased treatment regimens containing artemisinin, was used as a proxy for the proportion of patients who would be treated with an artemisinin-based drug. However, drug regimens were sometimes distributed for purposes other than clinical treatment, including stand-by treatment and prophylaxis of high-risk populations during outbreaks. In the first years following artemisinin introduction in Vietnam in 1989 and 1990,^26^,^27^ usage of artemisinins may have been higher than suggested by the purchase proportions in these data (Thuan PD, Thanh NV, Hien TT, personal communication). High malaria incidence during this decade necessitated large-volume purchases of chloroquine and primaquine for *P. vivax* treatment, but low rates of blood-slide confirmation would lead the vast majority of clinical cases to be treated with the most efficacious treatment available at that time, namely, artemisinin.

An important covariate that was omitted from the models was lagged malaria incidence, as it would be expected that incidence in the year *t* – 1 would affect incidence in the year *t*. We omitted this variable for two reasons. First, it would be reasonable to assume that malaria case numbers at the end of a calendar year would not have a strong effect on malaria case numbers recorded in the middle of the following year in an area with strong seasonality, especially after a 4–5 month period of low or zero malaria transmission. Second, an attempt to decompose the correlation between lagged incidence and current incidence into the direct effect and the indirect effect through the intermediate variable of drug purchase patterns may require more temporally resolved data. A dynamic malaria transmission model should be considered to test for these potentially causal relationships. Future analyses of this kind should aim to assemble case data sets with aggregation at the level of week or month as this will allow for a more detailed analysis of potentially lagged effects. An additional benefit of using more temporally resolved data will be improved data quality for the ITN/IRS measures and an understanding of whether ITN/IRS campaigns are followed by long-term or short-term decreased incidence in Vietnam.

As discussed previously, artemisinin is believed to reduce population-level malaria incidence through rapid parasite clearance, low treatment failure rate, and some pharmacological activity against the transmission stages of the parasite, gametocytes.^4^,^5^,^8^ Gametocidal activity of artemisinin has been shown to be more powerful in clearing *P. falciparum* parasites than *P. vivax* parasites.^8^ Additionally, *P. vivax* infections tend to produce gametocytes before patients present symptoms, whereas *P. falciparum* infections tend to yield both symptoms and gametocytes around the same time.^8^,^15^,^16^ Therefore, case management with artemisinin is expected to have a larger impact on *P. falciparum* transmission than *P. vivax* transmission because treatment may more effectively clear infection before patients are infectious. Previous studies have considered slide-confirmed *P. vivax* infections as a quasi control in expectation that *P. falciparum* transmission would be more sensitive to increasing use of artemisinin in case management.^15^,^16^ However, we found that the associations between artemisinin use and incidence of slide-confirmed infections with either parasite species were not significantly different. This null finding could result from a lack of statistical power to detect a potentially small difference in artemisinin potency against *P. falciparum* compared with *P. vivax.*

These results support the hypothesis that artemisinin scale-up may be a powerful strategy for not only patient care but also transmission reduction. However, evidence of slower parasite clearance rates in the Greater Mekong Subregion may threaten the individual and population-level potency of artemisinin-based therapies.^28^–^31^ It is crucial to understand the true population-level impact of artemisinin drugs, properly manage their distribution, and prepare strategies to prevent and contain resistance.^32^,^33^ Results from Thailand,^15^,^16^,^34^ Zanzibar,^6^ and now Vietnam support the hypothesis that case management with artemisinin-based combination therapies is likely to be strongly associated with reduced malaria transmission.

## Supplementary Material

Supplemental Datas.

## Figures and Tables

**Table 1:** Percent change in malaria incidence associated with a 10% increase in the listed covariate; with 95% confidence intervals in parentheses

	Suspected malaria	Slide-confirmed malaria	*Plasmodium falciparum*	*Plasmodium vivax*
Proportion of regimens containing artemisinin	−32.3*** (−40.6, −22.8)	−28.1*** (−39.5, −14.6)	−26.0*** (−36.5, −13.8)	−32.8*** (−46.5, −15.7)
Proportion of population covered by ITN or IRS	−12.5 (−29.0, +7.8)	−11.6 (−34.4, +19.1)	−2.7 (−23.7, +24.0)	−32.5 (−56.3, +4.4)
Proportion of population living in urban settings	−5.1 (−22.7, +16.5)	+4.0 (−5.3, +14.2)	+0.8 (−6.7, +8.9)	+15.2 (−7.7, +43.9)

*** *P* < 0.001.

ITN = insecticide-treated net; IRS = indoor residual spraying.

**Table 2:** Percent change in malaria incidence associated with a 10% increase in the listed covariate; with 95% confidence intervals in parentheses

	Suspected malaria	Slide-confirmed malaria	*Plasmodium falciparum*	*Plasmodium vivax*
Proportion of regimens containing artemisinin	−32.9*** (−39.8, −25.3)	−29.1*** (−41.0, −14.8)	−26.3*** (−37.2, −13.5)	−34.7*** (−47.2, −19.1)
Proportion of population covered by ITN or IRS	−8.2 (−23.7, +10.5)	−9.2 (−31.2, +19.9)	−0.9 (−22.1, +25.9)	−27.4 (−49.0, +3.4)
Proportion of population living in urban settings	−19.1 (−36.7, +3.3)	−5.4 (−18.9, +10.3)	−4.0 (−13.9, +6.9)	−8.1 (−31.8, +23.7)
Discretionary budget per capita	−10.8*** (−15.4, −6.0)	−8.2 (−17.7, +2.4)	−5.1 (−12.0, +2.3)	−16.1* (−28.1, −2.1)
Number of staff trained per capita	−14.1† (−26.3, +0.1)	−15.4 (−31.0, +3.8)	−13.6 (−28.6, +4.5)	−21.0 (−44.3, +12.1)

† *P* = 0.052.

* *P* < 0.05.

*** *P* < 0.001.

ITN = insecticide-treated net; IRS = indoor residual spraying.
