# Supplementary material for: Measuring the Association between Artemisinin-Based Case Management and Malaria Incidence in Southern Vietnam, 1991–2010
Source: Am J Trop Med Hyg. 2015 Apr 1;92(4):811–7. doi: 10.4269/ajtmh.14-0461 (PMC4385779; doi:10.4269/ajtmh.14-0461)
Supplement: Supplementary file 1 [file SD6.pdf]

SUPPLEMENTAL INFORMATION

In the main text, a Poisson model was fit using generalized estimating equations (GEE) to produce population-averaged associations between the independent variables and each malaria incidence outcome. Here, we allow for interaction between province and the main covariate, the proportion of treatment regimens that contain an artemisinin component, to measure province-specific associations between the main covariate and malaria incidence.

Using these models, we found that the proportion of treatment regimens that contain an artemisinin component was negatively associated with malaria incidence in all study provinces (Supplemental Figure 3). The magnitude of the population-averaged associations fall within the range of the province-specific associations for each of the four malaria outcomes tested: 1) clinically diagnosed suspected malaria cases, 2) slide-confirmed malaria cases, 3) slide-confirmed *Plasmodium falciparum* cases, 4) slide-confirmed *Plasmodium vivax* cases.

Despite a loss of statistical power caused by the use of many extra degrees of freedom, these significant results support the strength of the population-averaged associations reported in the main text. In addition to increased interpretability, the authors prefer the population-averaged measure of association because in theory we expect the effect of each intervention to have a similar magnitude in each province. Furthermore, strong correlations in both time and space for an infectious disease like malaria make it difficult to interpret more from the province-specific regression outputs than the general protective pattern observed.

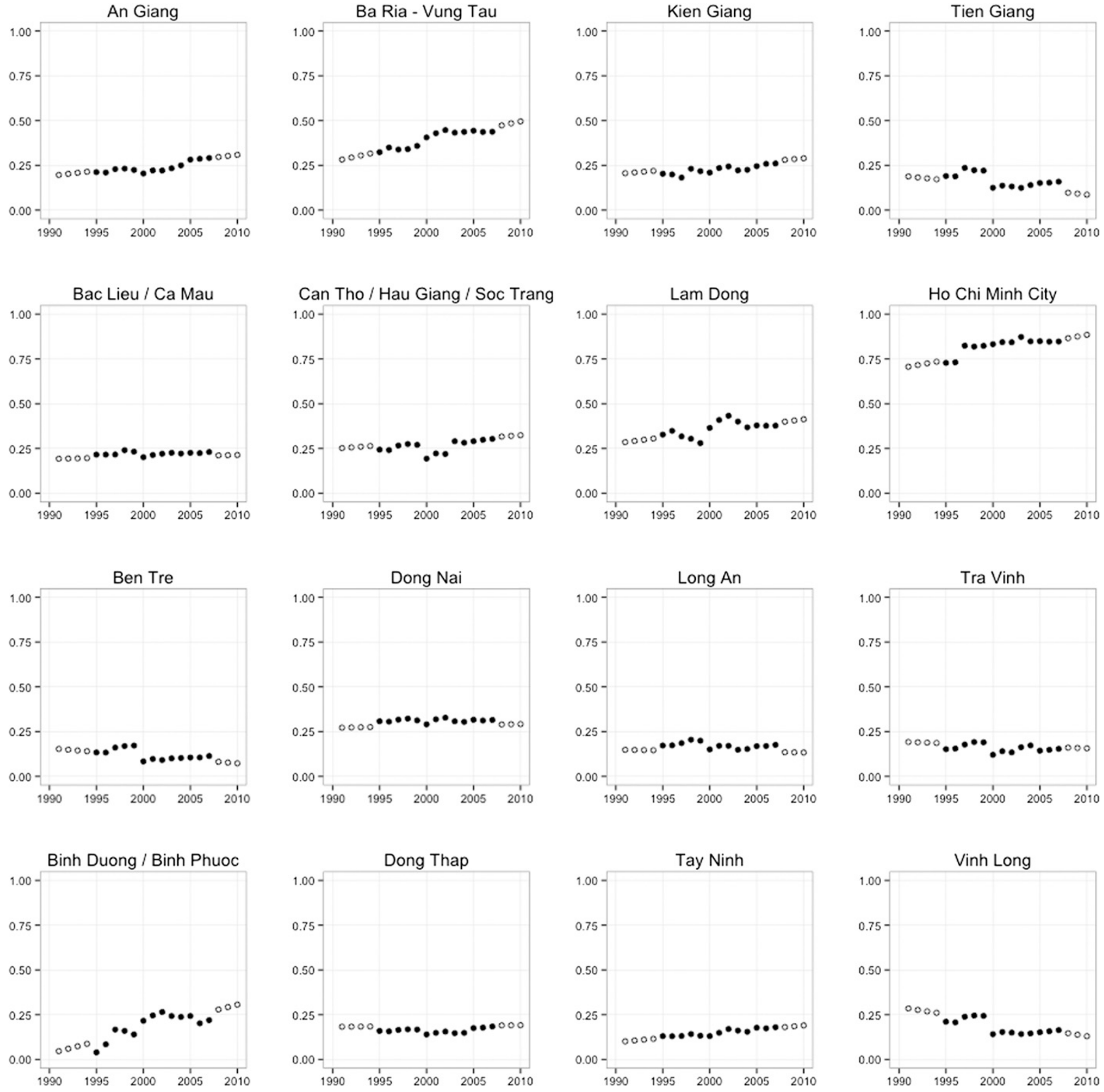

Supplemental Figure 1. Proportion of provincial population reported to be living in urban settings used in the analysis include observed (filled circles) and imputed (open circles) values. Observations from years 1995–2007 were fit with a linear regression and predicted values for years 1991–1994 and 2008–2010 were used to impute missing data.

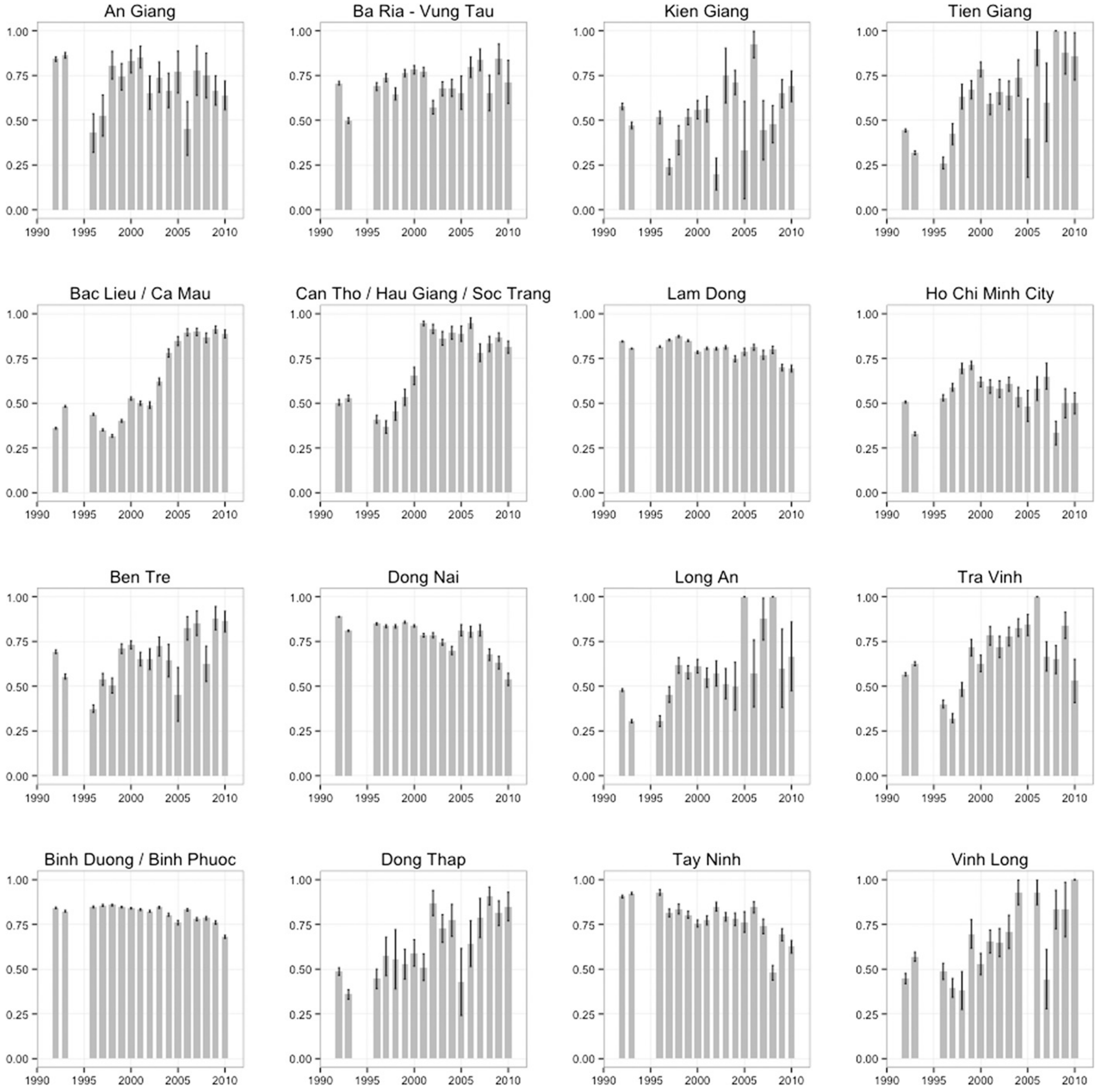

Supplemental Figure 2. Bar height indicates the proportion of slide-confirmed malaria cases that are infected with *Plasmodium falciparum* for each province by year. Error bars reflect standard error about each measure. The IMPE provincial malaria reports were unavailable for smear analysis for years 1991 and 1994–1995.

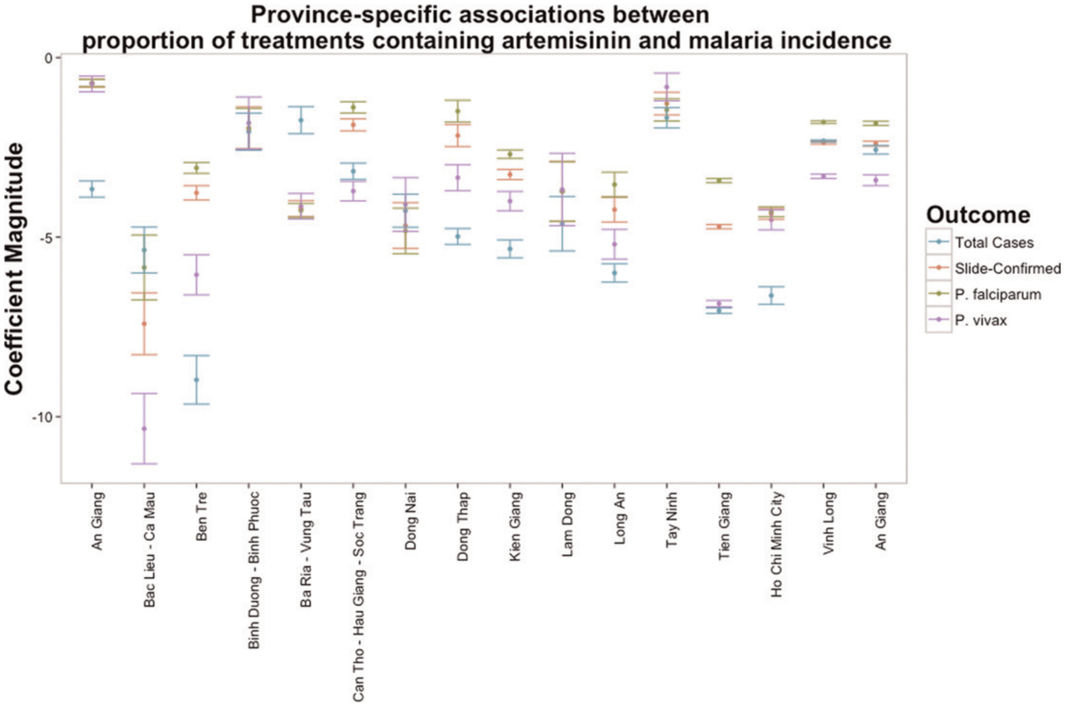

Supplemental Figure 3. All province-specific associations were significant between artemisinin use and each of the four malaria incidence outcomes. Population-averaged coefficients are indicated with the dotted lines. Results shown are derived from models including the proportion of drugs containing an artemisinin component, proportion of provincial population covered by insecticide-treated nets (ITNs) or indoor residual spraying (IRS), and proportion of provincial population living in urban settings. The results were qualitatively unchanged after the addition of the health systems capacity covariates: discretionary provincial malaria budget per capita and number of staff trained per 100 people.

Supplemental Table 1

Results from Spearman rank correlation and simple linear regression tests of changes in the proportion of regimens containing artemisinin over time*

	Proportion of regimens containing artemisinin			
	Spearman rank correlation test		Simple linear regression	
Province	ρ	*P*-value	beta	*P*-value
An Giang	0.732	0.002	0.0438	0.001
Bac Lieu/Ca Mau	0.885	< 0.001	0.0405	< 0.001
Ben Tre	0.861	< 0.001	0.0559	< 0.001
Binh Duong/ Binh Phuoc	0.932	< 0.001	0.0579	< 0.001
Ba Ria-Vung Tau	0.525	0.057	0.0360	< 0.001
Can Tho/Hau Giang/Soc Tran	0.903	< 0.001	0.0642	< 0.001
Dong Nai	0.924	< 0.001	0.0488	< 0.001
Dong Thap	0.872	< 0.001	0.0614	< 0.001
Kien Giang	0.821	< 0.001	0.0390	< 0.001
Lam Dong	0.929	< 0.001	0.0556	< 0.001
Long An	0.732	0.003	0.0326	< 0.001
Tay Ninh	0.885	< 0.001	0.0461	< 0.001
Tien Giang	0.775	0.001	0.0356	< 0.001
Ho Chi Minh City	0.809	< 0.001	0.0345	< 0.001
Tra Vinh	0.775	0.001	0.0649	< 0.001
Vinh Long	0.828	< 0.001	0.0582	< 0.001

*Trend tests elapse from 1992 to 2010.

Supplemental Table 2

Results from Spearman rank correlation and simple linear regression tests of changes in the proportion of provincial population protected by ITN or IRS over time*

	Proportion of population protected by ITN or IRS			
	Spearman rank correlation test		Simple linear regression	
Province	ρ	*P*-value	beta	*P*-value
An Giang	–0.157	0.576	–0.0004	0.351
Bac Lieu/Ca Mau	0.821	< 0.001	0.0173	< 0.001
Ben Tre	0.657	0.002	0.0041	0.003
Binh Duong/ Binh Phuoc	–0.398	0.083	–0.0093	0.097
Ba Ria-Vung Tau	–0.420	0.067	–0.0038	0.183
Can Tho/Hau Giang/Soc Tran	0.506	0.048	0.0030	0.001
Dong Nai	–0.202	0.393	–0.0026	0.478
Dong Thap	–0.543	0.039	–0.0021	0.102
Kien Giang	0.325	0.162	0.0038	0.031
Lam Dong	–0.564	0.011	–0.0131	0.013
Long An	–0.819	< 0.001	–0.0049	< 0.001
Tay Ninh	0.189	0.422	0.0008	0.657
Tien Giang	0.006	0.987	–0.0004	0.531
Ho Chi Minh City	–0.850	< 0.001	–0.0009	< 0.001
Tra Vinh	0.684	0.002	0.0033	< 0.001
Vinh Long	–0.065	0.814	0.0003	0.793

*Trend tests elapse from 1991 to 2010.
ITN = insecticide-treated net; IRS = indoor residual spraying.

Supplemental Table 3

Results from Spearman rank correlation and simple linear regression tests of changes in the proportion of provincial population living in urban settings over time*

		Proportion of population living in urban settings			
		Spearman rank correlation test		Simple linear regression	
		ρ	*P*-value	beta	*P*-value
Province	An Giang	0.775	0.003	0.0064	< 0.001
	Bac Lieu/Ca Mau	0.368	0.217	0.0006	0.413
	Ben Tre	–0.440	0.135	–0.0045	0.039
	Binh Duong/ Binh Phuoc	0.665	0.016	0.0135	0.002
	Ba Ria-Vung Tau	0.874	< 0.001	0.0111	< 0.001
	Can Tho/Hau Giang/Soc Tran	0.637	0.022	0.0046	0.072
	Dong Nai	0.027	0.935	0.0002	0.744
	Dong Thap	0.258	0.394	0.0012	0.256
	Kien Giang	0.852	< 0.001	0.0051	< 0.001
	Lam Dong	0.632	0.024	0.0065	0.037
	Long An	–0.462	0.115	–0.0017	0.196
	Tay Ninh	0.929	< 0.001	0.0046	< 0.001
	Tien Giang	–0.495	0.089	–0.0063	0.025
	Ho Chi Minh City	0.879	< 0.001	0.0087	0.002
	Tra Vinh	–0.280	0.353	–0.0014	0.391
	Vinh Long	–0.511	0.078	–0.0075	0.007

*Trend tests elapse from 1995 to 2007.
